# Supplementary material for: Intermediate Molecular Phenotypes to Identify Genetic Markers of Anthracycline-Induced Cardiotoxicity Risk
Source: Cells. 2023 Jul 27;12(15):1956. doi: 10.3390/cells12151956 (PMC10417374; doi:10.3390/cells12151956)
Supplement: Supplementary file 1 [file cells-12-01956-s001.zip › Supplementary Figures_S1-S4.pdf]

Figure S1

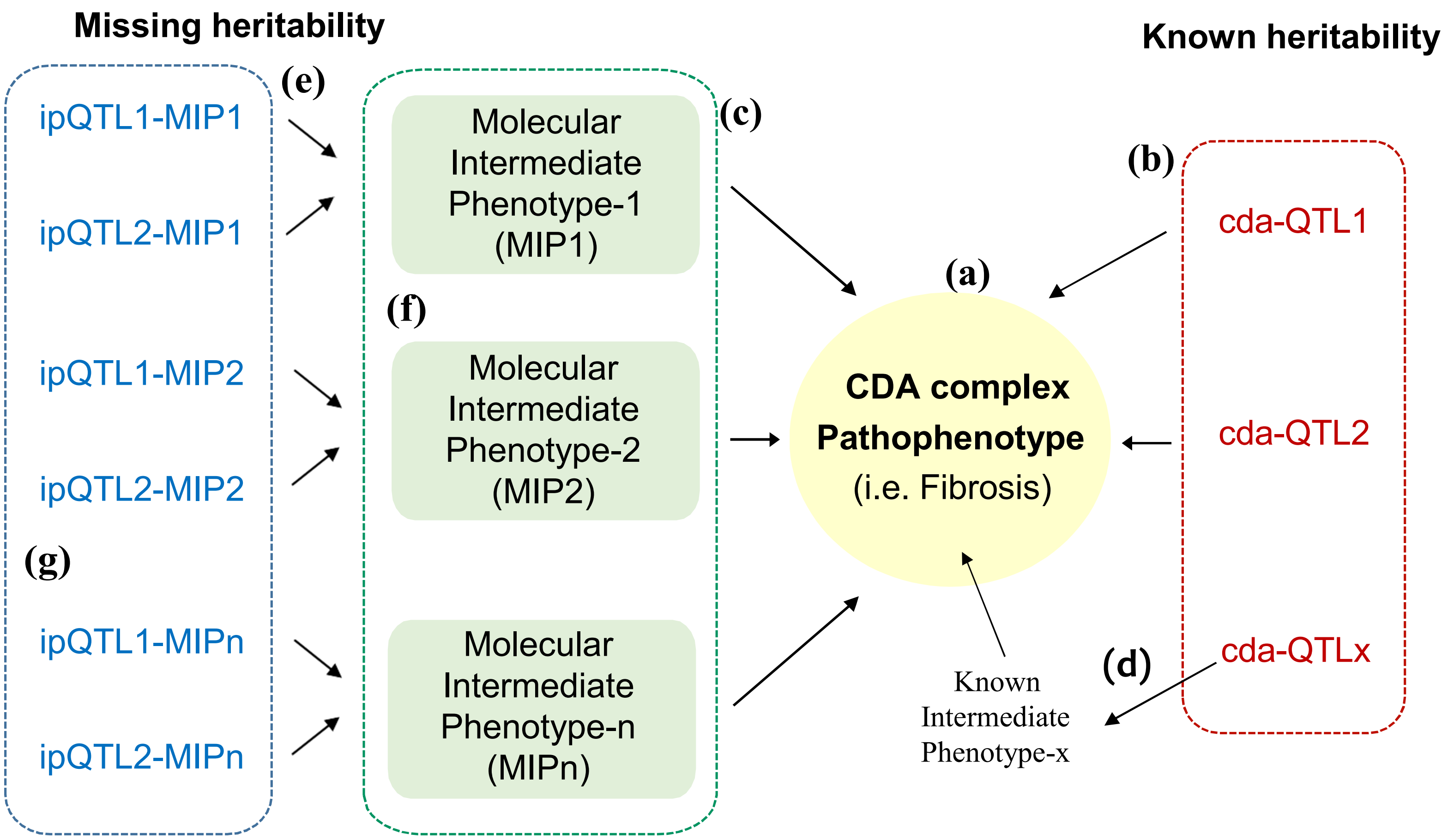

Missing heritability
Known heritability
(e)
(c)
(b)
(a)
(f)
(g)
(d)
ipQTL1-MIP1
ipQTL2-MIP1
ipQTL1-MIP2
ipQTL2-MIP2
ipQTL1-MIPn
ipQTL2-MIPn
Molecular Intermediate Phenotype-1 (MIP1)
Molecular Intermediate Phenotype-2 (MIP2)
Molecular Intermediate Phenotype-n (MIPn)
CDA complex Pathophenotype (i.e. Fibrosis)
Known Intermediate Phenotype-x
cda-QTL1
cda-QTL2
cda-QTLx

Figure S1. Graphical Representation of the Approach. Genetic determinants linked to intermediate phenotypes may elucidate the missing heritability of a complex trait. (a.) The rationale for this study is that cardiotoxicity due to anthracyclines (CDA) is a complex trait. **(b.)** Consequently, CDA phenotypic variation and risk are determined by numerous common genes, each with a minor effect. The genes associated with the variation of the complex trait (the known heritability) are located in regions called quantitative trait loci (QTLs), referred to here as cda-QTLs. **(c.)** Numerous intermediate phenotypes are involved in the pathogenesis of complex traits such as CDA, which also influences its phenotypic variation. **(d.)** Indeed, cda-QTLs directly linked to the phenotypic variation of CDA also act through intermediate phenotypes (not shown), only some of which would be known. The cda-QTLs are simultaneously linked to intermediate phenotypes and the main trait via a process called mediated pleiotropy. **(e.)** However, most of the genetic components of complex traits remain undetected and form part of the missing heritability, the origin of which continues to be a subject of debate. Nonetheless, genetic determinants linked to intermediate phenotypes (ip) could contribute to the heritability of the complex trait (here ipQTLs). Those ipQTLs that cannot be detected as being directly linked at the CDA level as mediated pleiotropy would be responsible for part of the missing heritability and may partially account for the phenotypic variation and risk of a complex trait (CDA, in this case). **(f.)** The simplest intermediate phenotypes are those of molecular origin. In this study, we selected molecules associated with cardiotoxicity identified in previous studies. **(g.)** In our study, another series of ipQTLs were linked to intramyocardial levels of intermediate molecular phenotypes. These ipQTLs are not directly linked to CDA because their effect is diluted due to their low potency, and we propose that they would contribute to the missing heritability of CDA as a complex trait.

Figure S2

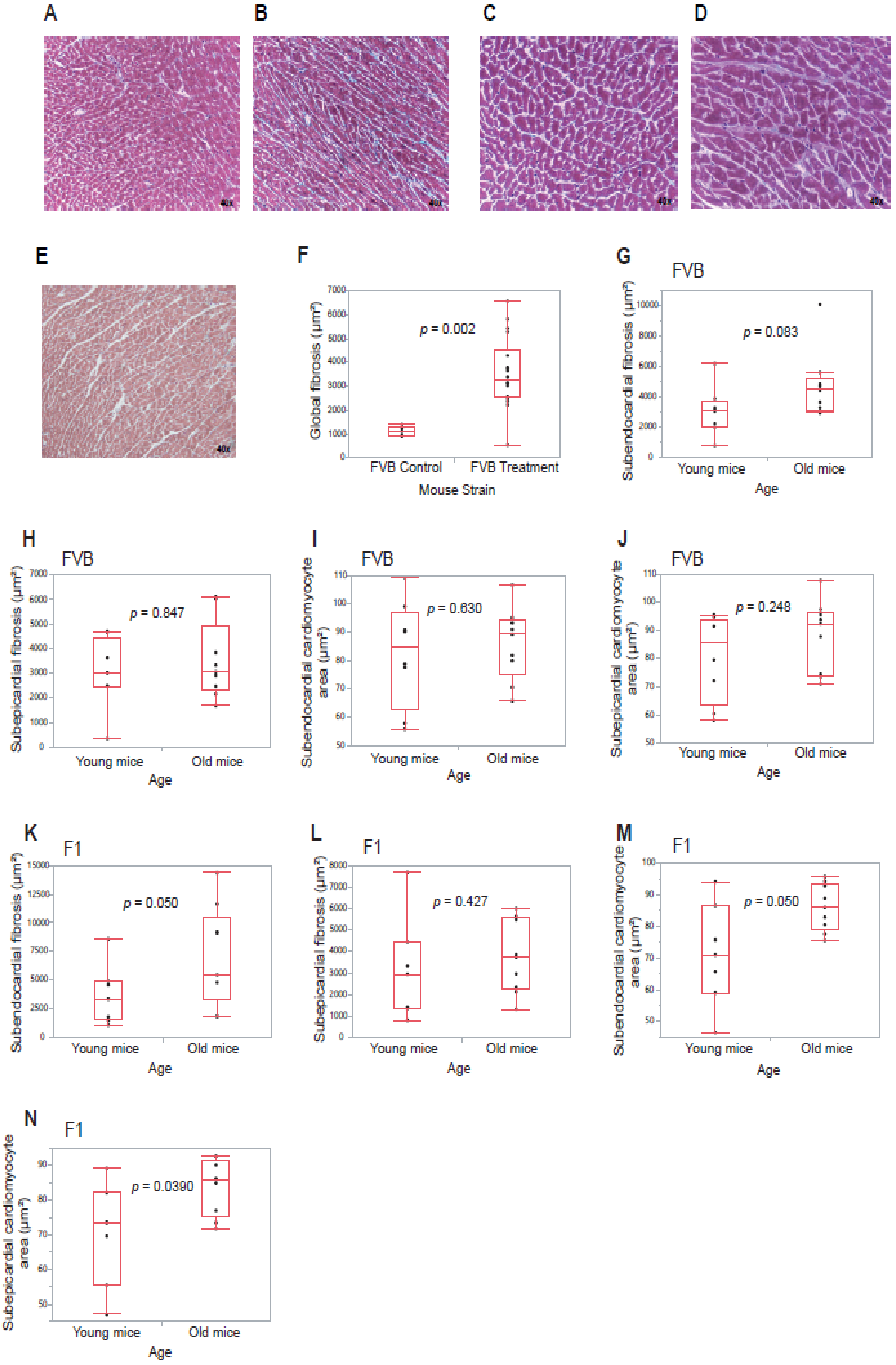

A
B
C
D
E
F
Global fibrosis (µm²)
p = 0.002
FVB Control
FVB Treatment
Mouse Strain
G
FVB
Subendocardial fibrosis (µm²)
p = 0.083
Young mice
Old mice
Age
H
FVB
Subepicardial fibrosis (µm²)
p = 0.847
Young mice
Old mice
Age
I
FVB
Subendocardial cardiomyocyte area (µm²)
p = 0.630
Young mice
Old mice
Age
J
FVB
Subepicardial cardiomyocyte area (µm²)
p = 0.248
Young mice
Old mice
Age
K
F1
Subendocardial fibrosis (µm²)
p = 0.050
Young mice
Old mice
Age
L
F1
Subepicardial fibrosis (µm²)
p = 0.427
Young mice
Old mice
Age
M
F1
Subendocardial cardiomyocyte area (µm²)
p = 0.050
Young mice
Old mice
Age
N
F1
Subepicardial cardiomyocyte area (µm²)
p = 0.0390
Young mice
Old mice
Age

Figure S2. Varying Degrees of Cardiac Histopathological Damage Post-Chemotherapy. A-D) Anthracycline chemotherapy. **E**) No significant histopathological alterations were observed in the absence of chemotherapy—Masson-Goldner Trichrome staining 40X. **F**) Quantification of the area of cardiac fibrosis in BVF mice treated and not treated with doxorubicin. G-N) Comparison of CDA grade between mice of the same strain, FVB or F1, based on age, with a separation between young and old mice using the median. The following comparisons are presented: subendocardial fibrosis area in FVB (G); subepicardial fibrosis area in FVB (H); cardiomyocyte area in the subendocardial zone in FVB (I); cardiomyocyte area in the subepicardial zone in FVB (J); subendocardial fibrosis area in F1 (K); subepicardial fibrosis area in F1 (L); cardiomyocyte area in the subendocardial zone in F1 (M); cardiomyocyte area in the subepicardial zone in F1 (N). Mann-Whitney U test was performed. Panels G-N, N = 7 mice per group were compared.

Figure S3

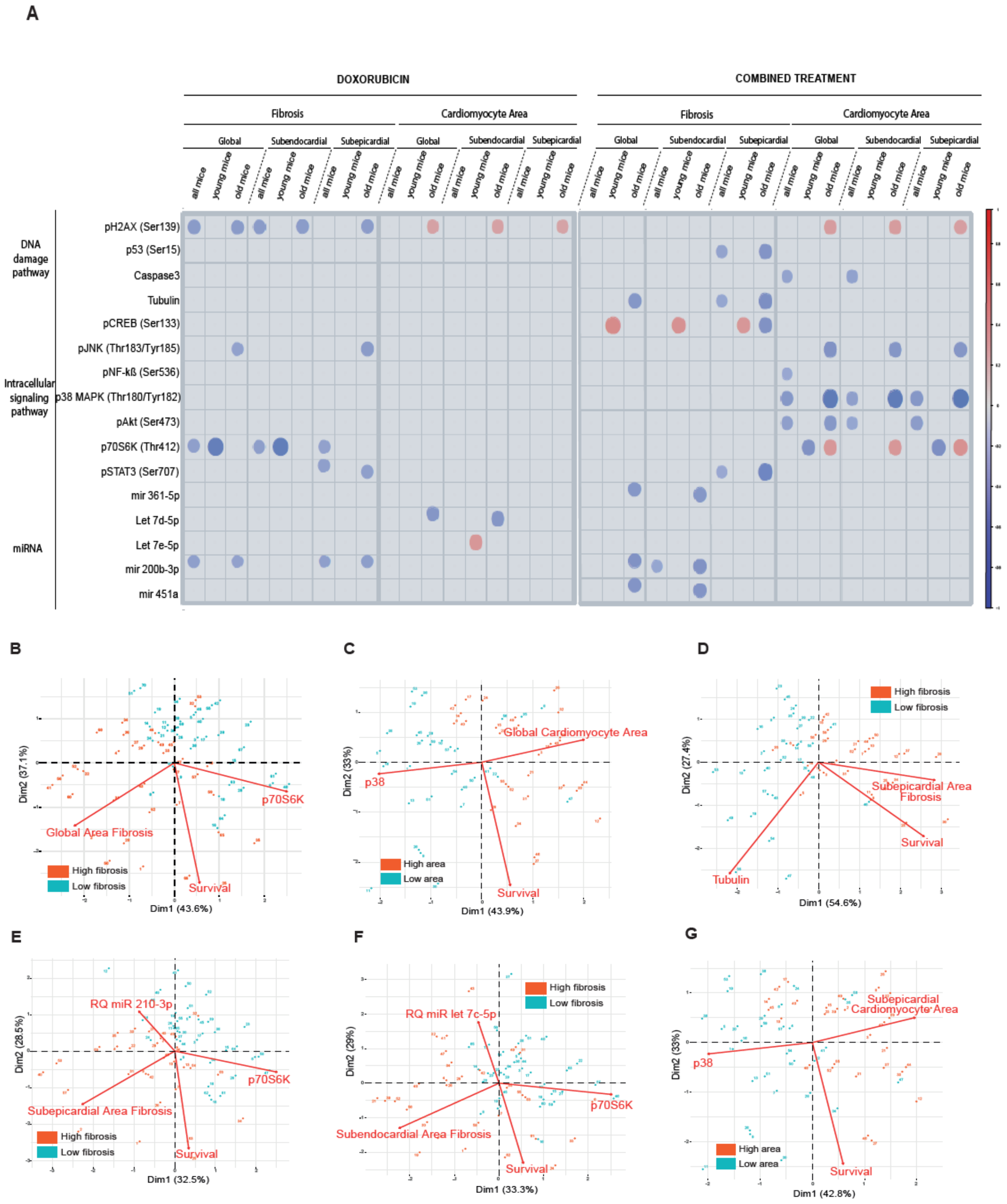
A
DOXORUBICIN
COMBINED TREATMENT
Fibrosis
Cardiomyocyte Area
Global
Subendocardial
Subepicardial
all mice
young mice
old mice
DNA damage pathway
Intracellular signaling pathway
miRNA
pH2AX (Ser139)
p53 (Ser15)
Caspase3
Tubulin
pCREB (Ser133)
pJNK (Thr183/Tyr185)
pNF-kß (Ser536)
p38 MAPK (Thr180/Tyr182)
pAkt (Ser473)
p70S6K (Thr412)
pSTAT3 (Ser707)
mir 361-5p
Let 7d-5p
Let 7e-5p
mir 200b-3p
mir 451a
B
Global Area Fibrosis
p70S6K
Survival
High fibrosis
Low fibrosis
Dim1 (43.6%)
Dim2 (37.1%)
C
Global Cardiomyocyte Area
p38
Survival
High area
Low area
Dim1 (43.9%)
Dim2 (33%)
D
High fibrosis
Low fibrosis
Subepicardial Area Fibrosis
Survival
Tubulin
Dim1 (54.6%)
Dim2 (27.4%)
E
RQ miR 210-3p
p70S6K
Subepicardial Area Fibrosis
Survival
High fibrosis
Low fibrosis
Dim1 (32.5%)
Dim2 (28.5%)
F
High fibrosis
Low fibrosis
RQ miR let 7c-5p
p70S6K
Subendocardial Area Fibrosis
Survival
Dim1 (33.3%)
Dim2 (29%)
G
Subepicardial Cardiomyocyte Area
p38
Survival
High area
Low area
Dim1 (42.8%)
Dim2 (33%)

Figure S3. Association of Intermediate Molecular Phenotypes with Anthracycline-Induced Cardiotoxicity (CDA). A) A correlogram is presented to display the degree of correlation between CDA-related molecular subphenotypes under various conditions. The magnitude and color intensity of each circle (refer to scale bar) signify the value of the Spearman correlation coefficient. Only correlations with a corresponding P-value < 0.05 are depicted. The numeric results are documented in Supplementary Table S3. **B-G**) Intermediate Molecular Phenotypes are Associated with Cardiotoxicity Due to Anthracyclines (CDA). Principal component analyses classify mice with high and low CDA susceptibility based on the myocardium's levels of intermediate molecular phenotypes under different conditions. Intermediate molecular phenotypes were selected by multiple regression analyses. Only those multivariate models for the whole cohort of mice described in Table S4 are included. The models are also indicated in Table S4. Fibrosis after doxorubicin therapy (model-1) (B). Cardiomyocyte area after combined therapy (model-15) (C). Fibrosis in the subepicardial area after combined therapy (model-12) (D). Fibrosis in the subendocardial area after doxorubicin (model-7) (E). Fibrosis in the subepicardial area after doxorubicin (model-4) (F). Subepicardial cardiomyocyte area after combined therapy (model-18) (G).

Figure S4

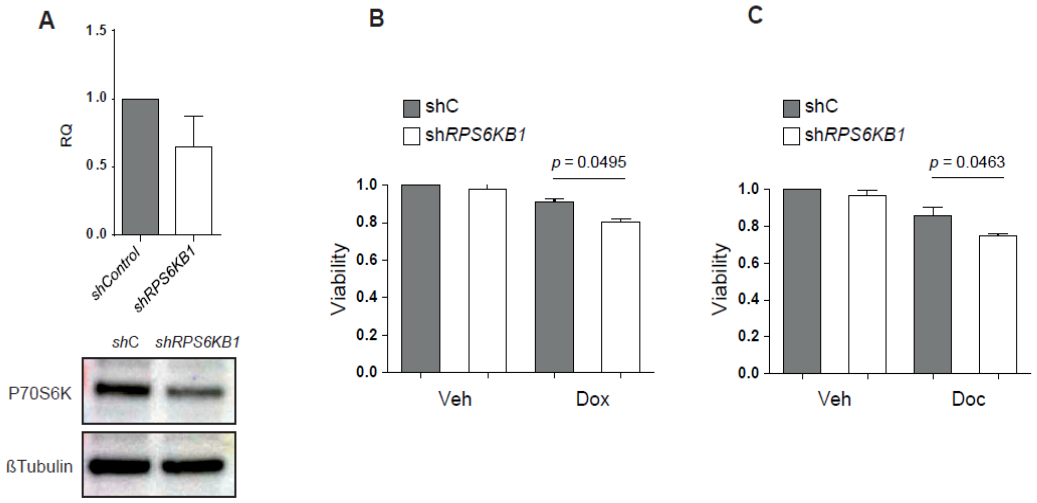
A
1.5
1.0
0.5
0.0
RQ
shControl
shRPS6KB1
shC
shRPS6KB1
P70S6K
ßTubulin
B
shC
shRPS6KB1
p = 0.0495
Viability
1.0
0.8
0.6
0.4
0.2
0.0
Veh
Dox
C
shC
shRPS6KB1
p = 0.0463
Viability
1.0
0.8
0.6
0.4
0.2
0.0
Veh
Doc

Figure S4. Downregulation of RPS6KB1 Levels in Human-Induced Pluripotent Stem Cell-Derived Cardiomyocytes (hi-PSC-CMs). A) RPS6KB1 levels were downregulated in human-induced pluripotent stem cell-derived cardiomyocytes (hiPSC-CMs) using a specific smart pool containing three siRNAs. **B, C**) Cell viability decreased after treatment with doxorubicin (B) or docetaxel (C). The experiment was repeated in two sets of hiPSC-CMs. In each experiment, cells were infected with lentivirus in triplicate. The panels show the mean ± SEM. Mann–Whitney U test was used.
