# Supplementary material for: Intermediate Molecular Phenotypes to Identify Genetic Markers of Anthracycline-Induced Cardiotoxicity Risk
Source: Cells. 2023 Jul 27;12(15):1956. doi: 10.3390/cells12151956 (PMC10417374; doi:10.3390/cells12151956)
Supplement: Supplementary file 1 [file cells-12-01956-s001.zip › Supplementary_Methods_.pdf]

Supplementary Material and Methods

Intermediate molecular phenotypes to identify genetic markers of anthracycline-induced cardiotoxicity risk

Index

-Supplementary Methods

1. Protocols related to the quantification of intermediate molecular phenotypes p.S2

2. Mouse genotyping…………………………………………………………………...p.S3

3.Human-induced pluripotent stem cell-derived cardiomyocytes (hi-PSC-CMs) protocols……………………………………………………………………………...p.S4

-References...p. S6

Supplementary Methods

1Protocols related to the quantification of intermediate molecular phenotypes

1.1 Protein extraction

Approximately 10-15 mg of frozen cardiac tissue were homogenized using the FastPrep Homogenizer system (FP120, Bio 101 Thermo Savant) and ceramic beads (Precellys Lysing Kit CkMix, Precellys) in lysis buffer (Lysis Buffer 1X, Milliplex) to which a cocktail of protease inhibitors (Roche Complete Mini) and phosphatase inhibitors (PhosSTOP EASYpack, Roche) was added. The quantification of signaling proteins and other intermediate molecular phenotypes is described in the supplementary methods.

1.2. Protein quantification

Protein concentration was quantified using the BCA Protein Assay test, following the manufacturer's instructions. Each sample was analyzed in triplicate. Absorbance was measured using the Xfluor program. Measurements were taken from distinct samples in 96-well plates. These multiplex assays have the advantage over ELISA in that they allow the levels of various proteins to be quantified in a single assay. They are based on 6.45-µm-diameter magnetic microspheres internally stained with a mixture of two fluorescent dyes and a capture antibody attached to them.

1.3 Quantification of telomere length by QPCR

DNA was phenol-chloroform extracted using heart tissue previously frozen in liquid nitrogen and stored at -80°C. The concentration of double-stranded DNA (dsDNA) was measured at 260 nm by a spectrophotometer (Nanodrop ND-1000) in triplicate. PCR was performed following the conditions published elsewhere (Hastie et al. 1990). Briefly, PCR reactions were carried out in a total volume of 12 µl in Twin-Tec real-time PCR plates 96 (#0030132513, Eppendorf) with the following reagents: 6 µl of PerfeCTa SYBR® Green SuperMix ROX (#733-1188, VWR) (1X) and 1.6 µl of 150 mM forward and reverse telomeric primers (*5′*-

CGGTTTGTTTGGGTTTGGGTTTGGGTTTGGGTTTGGGTT-3' and *5'-GCTTGCCTTACCCTTACCCTTACCCTTACCCTTACCCT-3'*, respectively). The acidic ribosomal phosphoprotein PO (36B4) gene was used to normalize the DNA quantification. Forward and reverse primers for the *36B4* gene were *5'- ACTGGTCTAGGACCCGAGAAG-3'* and *5'-TCAATGGTGCCTCTGGAGATT-3'*, respectively, to which 1 µl of DNA (10 ng) and 3.4 µl double-distilled H_2O were added. QPCR was performed in triplicate under indicated conditions (Hastie et al. 1990). The reactions were carried out in an automated thermocycler (Mastercycler ep Realplex2, Eppendorf), and data were analyzed using the $2^{-\Delta\Delta Ct}$ method (Livak and Schmittgen 2001).

1.4 Total RNA isolation and cDNA synthesis

In accordance with the manufacturer's instructions, total RNA from heart tissues was isolated using the QIAGEN®kit (RNeasy®, CAT#74104). RNA concentrations were determined using a spectrophotometer (Nanodrop) and microfluidic chips (Agilent). cDNA was synthesized on a Fluidigm Platform using the following materials: 5 µL of Taq buffer 5x with dNTPs, 2 µL of Taq polymerase (Roche), 9 µL of free-RNAse water, and 4 µL of total RNA (20 ng/µL).

2. Mouse genotyping

Briefly, DNA was extracted from the tail by the phenol-chloroform method. DNA concentrations were measured with a Nanodrop ND-1000 Spectrophotometer, and the PicoGreen double-stranded quantification method (Molecular Probes, Thermo Fisher Scientific Inc., Waltham, MA USA) was used for genotyping. Genome-wide scanning was carried out. Thus, the Illumina Mouse Medium Density Linkage Panel Assay was used for genotyping 130 F1BX mice at 1449 single nucleotide polymorphisms (SNPs). Genotypes were classified as FVB/FVB (F/F) or FVB/C57BL/6 (F/B). Ultimately, 806 SNPs were informative from the FVB and C57BL/6 mice; the average genomic distance between these SNPs was 9.9 Mb. The genotype proportion among the F1BX mice was normally distributed.

3. Human-induced pluripotent stem cell-derived cardiomyocytes (hi-PSC-CMs) protocols

3.1 hiPSC-derived cardiomyocytes protocol

Human-induced pluripotent stem cell-derived cardiomyocytes (hi-PSC-CMs) were generated following a protocol adapted from a previously published study (Herron *et al.* 2016). hi-PSC-CMs were generated by directed differentiation, thereby modulating Wnt/β-catenin signaling (Lian et al. 2012; Lian et al. 2013). hiPSC colonies were cultured on Matrigel-coated (Corning, 100 μg/mL) 6-well plates for 7 days using StemMACs iPSC Brew XF medium (Miltenyi Biotec). The medium was replaced every day. After that, hiPSCs were dissociated using 1 mL/well Versene solution (Life Technologies) at 37 °C for 5 min and reseeded as monolayers on Matrigel-coated 6-well plates in StemMACs iPSC Brew XF medium. After two days, on day 0 of differentiation, when monolayers reached 90% confluence, cells were washed with HBSS calcium, magnesium, no phenol red (Gibco). The medium was changed to RPMI supplemented with B27 minus insulin (Gibco) containing 12 μM CHIR99021 (Miltenyi Biotec). On day 1, the medium was changed to RPMI supplemented with B27 minus insulin. On day 3, the medium was changed to RPMI supplemented with B27 minus insulin, containing 5 μM of the small molecule Wnt inhibitor IWP4 (Stemgent). On day 5, the medium was changed to RPMI supplemented with B27 minus insulin. Finally, the medium was changed from the seventh day onwards to RPMI supplemented with B27 complete supplement, RPMI +B27 media (Gibco).

On day 31 of directed differentiation, cell cultures were trypsinized, and cardiomyocytes were purified using magnetically activated cell sorting to target and deplete non-myocytes from the cell population, which resulted in cardiomyocytes enriched to ~98% purity (Miltenyi Biotec, PSC-derived cardiomyocyte isolation kit, human). hiPSC-CMs were washed with no calcium, no magnesium, no phenol red (Gibco) and dissociated using 1 mL of 0.25% Trypsin/EDTA (Gibco) per well. Next, 6 mL of EB20 media was added per well of dissociated cells; each well

was triturated and then transferred into a sterile 50-mL conical tube. The EB20 medium was composed of 80% DMEM/F12 (Gibco), 0.1 mM non-essential amino acids (Gibco), 1 mM L-glutamine (Gibco), 0.1 mM β-mercaptoethanol (Gibco), 20% fetal bovine serum (FBS, Corning) and 10 μM blebbistatin (Sigma-Aldrich). After collecting the cells, a 70 μm strainer was used. The cells were then transferred into a 15-mL conical tube and centrifuged at 1000 RPM for 5 min at room temperature. After removal of the supernatant, 2 mL of MACs buffer was added, followed by trituration. Cells were centrifuged again at 1000 RPM for 5 min at room temperature. The supernatant was aspirated, and 80 μL of MACs buffer was added to resuspend the pellet. Then, 20 μL of non-cardiomyocyte depletion cocktail (Biotin-conjugated) primary antibody was added, and the cells were gently triturated. After primary antibody incubation for 5 min on ice, 2 mL MACs buffer was added, and cells were gently triturated, followed by a 1000 RPM for 5 min at room temperature. The excess primary antibody was aspirated, and 80 μL of MACs buffer resuspended the pellet. Next, it was mixed with 20 μL of anti-biotin magnetic microbeads (secondary antibody) and incubated on ice for 10 min. After secondary antibody incubation, cells were mixed with 1 mL of MACs buffer. A 70-μm strainer was then used before purifying the cardiomyocyte and non-cardiomyocyte populations using the autoMACS Pro Separator. Next, the purified iPSC-CMs were centrifuged, and the supernatant was aspirated. The purified hiPSC-CMs fractions were resuspended in EB20 media, plating 200k hiPSC-CMs per well in a 12-well plate with Matrigel-coated (Corning) for 15 days.

3.2 hiPSC-CMs lentiviral infection

HiPSC-CMs ($1x10^5$ cells/well) were seeded on a 24-well plate for three days and incubated in 250 μl OPTI-MEM medium (Gibco) for 2 hours before lentiviral infection. For the infection, 1 μl of *shControl* (Santa Cruz Biotechnology, Cat # sc-108080) or *shRPS6KB1* (Santa Cruz Biotechnology Cat # sc-36165-V) lentiviral particles were mixed in 50 μl OPTI-MEM medium in the presence of 1 μl polybrene (5 mg/ml). The mixture was incubated for 30 minutes at room temperature and added to each well. The cells were incubated for 4 hours before the medium was changed. Downregulation of the *RPS6KB1* was evaluated by QPCR and quantified by the 2^-

$^{\Delta\Delta CT}$ method, and the levels of P70S6K by western blot (#9202 Cell Signaling). The drugs (Dox 750 nM for 24 hours and Doc 2.5 μg/ml for 8 hours) were added 48 hours post-infection, and crystal violet staining was used to analyze cell viability. After that, the medium was removed, the culture was fixed with 4% PFA, and washed with PBS 1X thrice. Crystal violet solution (0.5 g crystal violet powder (Sigma-Aldrich) dissolved in 20% methanol) was added to each well and incubated for 30 minutes at room temperature. After incubation, the staining solution was removed, and each well was washed gently with tap water. The plate was left to dry at RT for 16 hours. Methanol was added to each well and measured at 570 nm.

References

Hastie ND, Dempster M, Dunlop MG, Thompson AM, Green DK, Allshire RC. 1990. Telomere reduction in human colorectal carcinoma and with ageing. *Nature* **346**: 866-868.

Herron TJ, Rocha AM, Campbell KF, Ponce-Balbuena D, Willis BC, Guerrero-Serna G, Liu Q, Klos M, Musa H, Zarzoso M et al. 2016. Extracellular Matrix-Mediated Maturation of Human Pluripotent Stem Cell-Derived Cardiac Monolayer Structure and Electrophysiological Function. *Circulation Arrhythmia and electrophysiology* **9**: e003638.

Lian X, Hsiao C, Wilson G, Zhu K, Hazeltine LB, Azarin SM, Raval KK, Zhang J, Kamp TJ, Palecek SP. 2012. Robust cardiomyocyte differentiation from human pluripotent stem cells via temporal modulation of canonical Wnt signaling. *Proceedings of the National Academy of Sciences of the United States of America* **109**: E1848-1857.

Lian X, Zhang J, Azarin SM, Zhu K, Hazeltine LB, Bao X, Hsiao C, Kamp TJ, Palecek SP. 2013. Directed cardiomyocyte differentiation from human pluripotent stem cells by modulating Wnt/beta-catenin signaling under fully defined conditions. *Nature protocols* **8**: 162-175.

Livak KJ, Schmittgen TD. 2001. Analysis of relative gene expression data using real-time quantitative PCR and the 2(-Delta Delta C(T)) Method. *Methods* **25**: 402-408.
